# Bone Turnover Marker Profiling and Fracture Risk in Older Women: Fracture Risk from Age 75 to 90

**DOI:** 10.1007/s00223-022-00996-8

**Published:** 2022-06-24

**Authors:** Kaisa K. Ivaska, Fiona E. McGuigan, Linnea Malmgren, Paul Gerdhem, Helena Johansson, John A. Kanis, Kristina E. Akesson

**Affiliations:** 1 Institute of Biomedicine, University of Turku, Turku, Finland; 2 Clinical and Molecular Osteoporosis Research Unit, Department of Clinical Sciences Malmö, Lund University, Malmö, Sweden; 3 Department of Geriatrics, Skåne University Hospital, Malmö, Sweden; 4 Department of Clinical Science Intervention and Technology, Karolinska Institutet, Stockholm, Sweden; 5 Department of Surgical Sciences and Department of Orthopaedics, Uppsala University, Uppsala, Sweden; 6 Centre for Metabolic Bone Diseases, University of Sheffield, Sheffield, UK; 7 Mary McKillop Institute for Health Research, Australian Catholic University, Melbourne, Australia; 8 Department of Orthopedics Malmö, Skåne University Hospital, S-21428 Malmö, Sweden

**Keywords:** Bone, Osteoporosis, Bone turnover markers, Fracture

## Abstract

**Purpose:**

A major challenge in osteoporosis is to identify individuals at high fracture risk. We investigated six bone turnover markers (BTMs) to determine association with specific fracture types; the time-frame for risk prediction and whether these are influenced by age at assessment.

**Methods:**

Population-based OPRA cohort (*n* = 1044) was assessed at ages 75, 80, 85 and fractures documented for up to 15 years. Six BTMs were analyzed at each time-point (N-terminal propeptide of type I collagen, PINP; total osteocalcin, OC; bone-specific alkaline phosphatase, BALP; C-terminal telopeptide of type I collagen, CTX; tartrate-resistant acid phosphatase 5b, TRAcP5b; urinary osteocalcin). Hazard ratios (HR) for any, major osteoporotic, vertebral and hip fractures were calculated as short (1, 2, 3 years) and long-term risk (5, 10, 15 years).

**Results:**

At 75 year, high CTX levels were associated with an increased risk of all fractures, including major osteoporotic fractures, across most time-frames (HRs ranging: 1.28 to 2.28). PINP was not consistently associated. Urinary osteocalcin was consistently associated with elevated short-term risk (HRs ranging: 1.83–2.72). Other BTMs were directionally in accordance, though not all statistically significant. BTMs were not predictive for hip fractures. Association of all BTMs attenuated over time; at 80 year none were associated with an increased fracture risk.

**Conclusion:**

CTX, urinary OC and TRAcP5b are predictive for fracture in a 1 to 3 year, perspective, whereas in the long-term or above age 80 years, BTMs appear less valuable. Resorption markers, particularly CTX, were more consistently associated with fracture risk than formation markers in the very elderly.

**Supplementary Information:**

The online version contains supplementary material available at 10.1007/s00223-022-00996-8.

## Introduction

Osteoporotic fractures are a considerable problem in health care due to the consequences both for the patient and the burden on health care; moreover numbers will increase as the population ages [1]. A major challenge is correctly identifying individuals at high risk for fragility fractures. Deranged bone turnover/remodeling results in structural changes, low bone density, decreased bone strength and ultimately fracture. Bone turnover can be assessed by proteins or fragments of proteins released during remodeling. Usually, bone formation and resorption are coupled, and bone turnover markers (BTMs) released from either process reflect overall bone turnover.

Over the years a number of markers for resorption and formation have been evaluated in relation to bone loss, fracture risk and drug efficacy. These markers capture different aspects of bone metabolism essential to our understanding of bone turnover and include, for bone resorption, C-terminal crosslinked telopeptides of type I collagen (CTX) and tartrate-resistant acid phosphatase 5b (TRAcP5b), as indicators of collagen degradation and osteoclast activity respectively. For bone formation, markers include N-terminal propeptide of type I collagen (PINP) as an indicator of collagen formation, bone-specific alkaline phosphatase (BALP), indicating osteoblast activity and potentially mineralization, and lastly, osteocalcin, a non-collagenous protein secreted by osteoblasts and a surrogate marker for bone turnover. In addition, urinary osteocalcin fragments (U-OC) which may originate from bone resorption or formation, and most likely mirroring bone turnover overall, have been evaluated [2, 3]. The International Osteoporosis Foundation (IOF) and International Federation of Clinical Chemistry (IFCC) have recommended two reference markers for research and monitoring therapy, CTX and PINP [4]. Meta-analysis has confirmed a significant, albeit modest, association between high levels of these markers and increased fracture risk [5].

While the existing studies by ourselves [6, 7] and others [8–10] have advanced the literature surrounding the role of bone turnover markers as a tool for fracture management, there is still a gap in our understanding of the association between BTMs and fracture risk particularly in older women in whom fractures are most prevalent, in part due to the heterogeneity in study design and reporting [11–19]. The present study was performed in the prospective, population-based OPRA cohort of women, all of whom were 75 years of age at inclusion, with re-investigation at ages 80 and 85 and fracture data with a 15 year perspective. The objective was to investigate the two IOF recommended BTMs in addition to four others reflecting different aspects of bone turnover. We aimed to determine the association of BTMs with specific fracture sites, the effect of time on risk prediction and the effect of age at the time of assessment.

## Materials and Methods

### Subjects

The Malmö Osteoporotic Prospective Risk Assessment (OPRA) cohort is a longitudinal population-based cohort of women age 75 years at invitation, who were randomly selected from the Malmö city files between 1995 and 1999. No exclusion criteria were applied [20]. Of the 1604 invited, 1044 (65%) attended baseline. Of these, 715 (69%) attended the 5 year follow-up at age 80 years and 382 (37%) attended the 10 year follow-up visit at age 85 years. Participants completed an extensive questionnaire on health, nutrition, medication and lifestyle [21]. Each visit also included measurements of bone mineral density (BMD), muscle strength and functional tests, all performed at the dedicated Osteoporosis Research Unit [21]. The study was approved by the Regional Ethical Review Board in Lund, Sweden. All participants gave written informed consent and the study was performed according to the principles of the Helsinki declaration.

### Fracture Registration

All fractures were recorded and verified through *x*-ray and medical files from the Department of Radiology [20]. Women with fractures sustained prior to study start (between the ages of 50 and 75 years were categorized as having a prior osteoporotic fracture [21]. Over half the women had an adult fracture prior to baseline (*n* = 534; 51.1%), most of them after the age of 50 (*n* = 478), primarily at an osteoporotic site (*n* = 322).

Prospective fractures (regardless of participation at follow-up) were recorded from inclusion until end of follow-up (31 October, 2012) or date of death, which was obtained from the national Swedish register of deaths as described elsewhere [21–23]. Vertebral fractures were captured as those which had been diagnosed and recorded and we additionally recorded the presence of vertebral fractures from available lateral chest or abdominal radiographs from unrelated investigations. In this analysis the mean follow-up is 15 years (range 13.4–17) corresponding to 12,163 person-years. As previously described in detail, loss of fracture information is exceptionally low [24]. Fractures were classified into hip, pelvis, vertebral, distal radius, proximal humerus, and ‘other fractures’; and also into major osteoporotic fractures as defined by FRAX i.e. hip, vertebral, distal radius and proximal humerus [25]. Fractures of the face, hands and feet or resulting from pathology or high energy (*n* = 141) were excluded.

### Bone Turnover Markers

Serum and urine samples were collected as previously described [7] at baseline, 5 year and 10 year follow-up visits. Briefly, non-fasting blood samples were obtained between 08:00 and 13:00; centrifuged and processed within 2 h after phlebotomy. Urine samples were obtained as first morning void. All samples were stored at − 80 °C. At the 10 year visit, a fasting sample was also collected; drawn on the same day approximately 2 h prior to the non-fasting sample.

Six bone turnover markers capturing different aspects of bone metabolism were assessed. All analyses were performed blinded and in duplicate.

CTX, C-terminal crosslinked telopeptides of type I collagen, was measured using Elecsys β-CrossLaps ELISA (Roche Diagnostics, Indianapolis, IN, USA). TRAcP5b, tartrate-resistant acid phosphatase 5b, was measured using BoneTRAP ELISA (IDS Ltd., Bolton, UK). PINP, intact N-terminal propeptide of type I collagen, was measured using IDS-iSYS Intact PINP assay (IDS Ltd, Bolton, UK). BALP, bone-specific alkaline phosphatase, was measured with Metra BAP Assay (Quidel, Corporation, San Diego, CA, USA). Osteocalcin, was measured as total osteocalcin (tOC) using a previously described two-site immunoassay which detects the N-terminal midsegment of the OC molecule [26, 27]. U-OC, was measured with a two-site assay for osteocalcin midfragment in urine [2], normalized for urinary creatinine. BTMs were assayed in-house, at the accredited clinical chemistry laboratory at Skåne University Hospital or by Pharmatest Services Ltd, Finland. The reported within-assay and total variations for the assays have been reported previously [2, 6, 26, 28].

At age 85 serum CTX was analyzed in both non-fasting and fasting samples and as expected, non-fasting CTX values were lower (median -36%, *p* < 0.0001). However, fasting and non-fasting CTX values (where both samples available, *n* = 336) correlated highly (*r* = 0.93, *p* < 0.0001; log-transformation; linear regression). *Supplemental* Fig. 1 . The majority of women (80%) were classified into similar tertiles regardless of fasting state.

The number of samples available and used for BTM analysis in this study were: 999–1024 (baseline); 667–696 (5 years) and 342–369 (10 years). OC and BALP were available only for baseline and 5 years.

### Statistical Analyses

Baseline descriptive data are presented as mean and SD or median and range. Change was assessed with paired *t*-test. Comparisons between women who sustained a fracture and fracture-free women were tested with Student’s *t*-test or Pearson chi-square. All BTMs were log transformed (Shapiro–Wilk test < 0.95) and also stratified into tertiles (Tertile_Low_, Tertile_Mid_. Tertile_High_) for the entire cohort (*n* = 1044) and separately for the *n* = 715 women who participated at *both* baseline and 5 year follow-up. For the latter, the combination of the two assessments (being in Tertile_High_ at 75 year *and* 80 year vs. Tertile_Low_ at both ages) was also used, as a proxy for long-term turnover status.

**Fig. 1:**
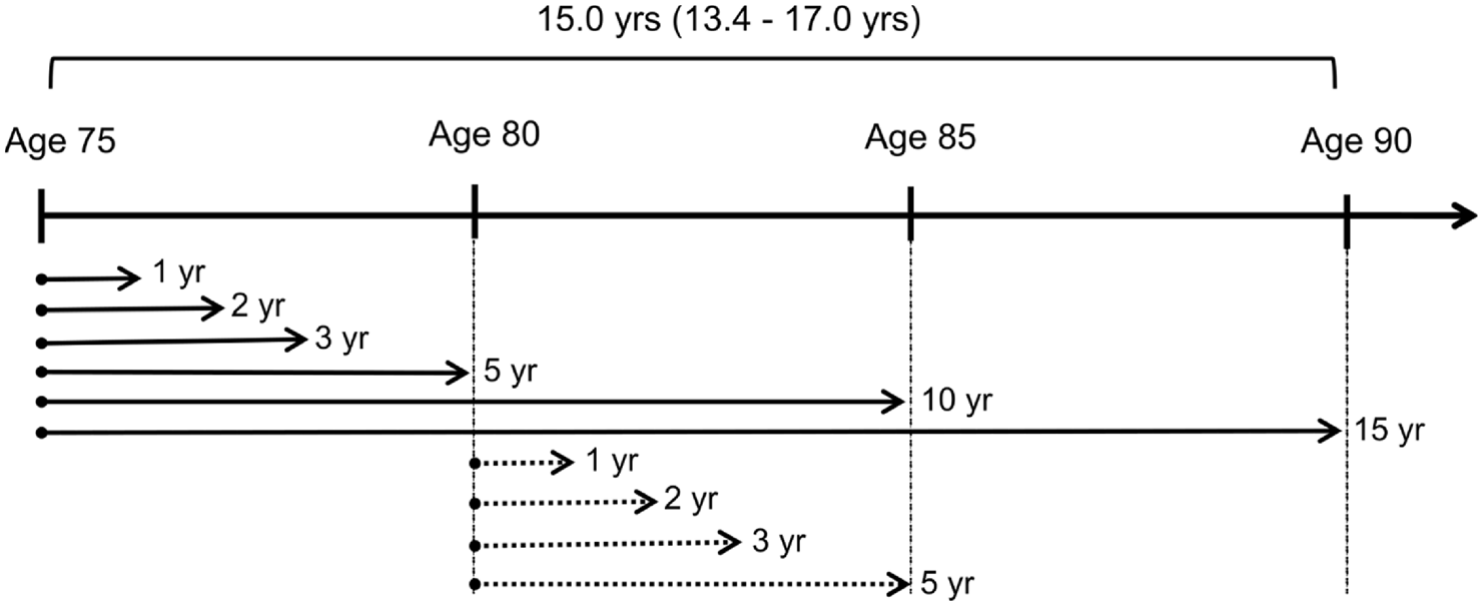
Analysis framework for determining fracture prediction by BTMs in the OPRA cohort Analyses were performed first using BTMs measured at baseline (age 75) and then BTMs measured at age 80. Fracture prediction was assessed as ‘short-term risk’ (1, 2 or 3 years) and ‘long-term risk’ (5, 10 or 15 years)

The primary outcome was the first incident fracture of any kind. Secondary outcomes were the first incident hip fracture (HFx), vertebral fracture (VFx) or major osteoporotic fracture (MOF, i.e. hip, vertebral, distal radius and proximal humerus fracture); compared with fracture-free women. The analysis strategies are illustrated in Fig. 1*.* Fracture prediction was assessed as ‘short-term risk’ (1, 2 or 3 years after BTM measurement) and as ‘long-term risk’ (5, 10 or 15 years). Analyses were performed, first using BTMs measured at age 75, and then using BTMs measured at age 80 years. Results are reported unadjusted *or/and* adjusted for smoking, bisphosphonate use and prior osteoporotic fracture (i.e. between 50 and 75 years of age). BMD was not adjusted for, except as an example, when additional adjustment for baseline total hip BMD was performed for 3 year risk of any fracture.

Kaplan–Meier survival analysis with log-rank testing was used to compare BTM Tertile_high_ vs Tertile_Low_. Endpoint was date of first fracture (or end of follow-up time, if fracture free) and date of death as censor. Risk was estimated by Cox proportional hazard ratios (HR, 95% confidence intervals); reference Tertile_Low_. Competing risk analysis using the Fine and Gray method was also performed for PINP and CTX and 3 year risk of any fracture. In a sub-analysis to facilitate comparison between BTMs, and with other studies, HRs per SD change in standardized BTM Z-score were calculated [29, 30].

To demonstrate the predictive value of bone turnover markers over time, fracture risk (HR per SD change in BTMs) was further analyzed as a function of follow-up time using a modification of the Poisson regression model [31, 32]. Follow-up used person-years, with each participant’s observation period divided into monthly intervals. *Time to fracture* counted only the first fracture after baseline. Time since baseline (continuous variable) and the association with fracture risk was described with spline functions using knots at 2, 5 and 10 years. Splines were second-order functions between breakpoints and linear functions at the tails, resulting in a smooth curve. Model-1 adjusted for age; Model-2 adjusted for age, smoking, bisphosphonate use and prior osteoporotic fracture. For BTMs measured at age 80, a simple interaction between time since baseline and CTX was used, due to reduced statistical power.

Statistical analyses used SPSS, v25 (SPSS, Inc., Chicago, IL, USA) and GraphPad Prism (8.1.2, GraphPad Software, San Diego, CA, USA). Neither BTMs nor fracture outcomes are fully independent, therefore applying Bonferroni correction would be over-stringent. We report uncorrected *p*-values (two-tailed), acknowledging that multiple tests were performed. Confidence intervals indicate the reliability of the associations and nominal significance was considered with *p* < 0.05.

## Results

During the study, 50.2% of the women (*n* = 524) sustained at least one fracture of any type (Supplemental Table 1). The average time to a first fracture was 6.4 years (range 0.06–15.3). A total of 1048 fractures was recorded, a frequency of 8.6% per person per year. During the first three years of follow-up, the cumulative number of women with fracture was 51 (4.9%) at 1 yr, 93 (8.9%) at 2 yr and 130 (12.5%) at 3 year.

The characteristics of the OPRA cohort at ages 75, 80 and 85 years are presented in Table 1*,* in addition to details of those who attended all three visits; BTMs are reported as original values. In terms of the dynamics of bone turnover markers in elderly women, most BTMs increased from age 75 to 80, subsequently decreasing at age 85. Baseline BTMs did not differ by fracture status, although CTX was marginally higher in women who fractured during the first 5 years of follow-up (*p* = 0.047, data not shown).

**Table 1:** Study population. Characteristics of (I) OPRA participants at age 75, 80 and 85; (II) those women attending all three visits

	Age 75		Age 80		Age 85		*p*-value*		
	*n* = 1044		*n* = 715		*n* = 382				
	Mean/Median	(SD)/(IQR)	Mean/Median	(SD)/(IQR)	Mean/Median	(SD)/(IQR)	75 year vs 80 year	75 year vs 85 year	80 year vs 85 year
(I) Cross-sectional, women attending EACH visit									
Age, years	75.2	(0.15)	80.2	(0.16)	85.2	(0.14)			
Weight, kg	67.8	(11.7)	66.1	(11.5)	64.0	(10.9)	< 0.001	< 0.001	< 0.001
Height, cm	160.5	(5.7)	159.1	(5.7)	158.4	(5.8)	< 0.001	< 0.001	< 0.001
BMI, kg/m^2^	26.3	(4.2)	26.1	(4.2)	25.4	(4.0)	0.001	< 0.001	< 0.001
Current smoker, n (%)	145	(14%)	76	(11%)	22	(6%)			
Vit D supplement, n (%)	65	(6%)	113	(16%)	97	(25%)			
Ca supplement, n (%)	69	(6%)	181	(25%)	161	(42%)			
Bisphosphonate, n (%)	33	(3%)	50	(7%)	44	(12%)			
CTX, ng/L	263	(174–397)	311	(213–431)	257	(192–366)	< 0.001	0.30	0.001
PINP, ng/mL	48.1	(36.7–62.1)	49.8	(37.1–63.8)	43.8	(31.9–56.6)	0.071	< 0.001	< 0.001
TRAcP5b, U/L	3.3	(2.6–4.0)	5.1	(4.1–6.7)	3.8	(3.0–4.6)	< 0.001	< 0.001	< 0.001
U-OC, µg/mmol	1.0	(0.7–1.6)	1.3	(0.8–1.9)	2.1	(1.3–2.9)	< 0.001	< 0.001	< 0.001
tOC, ng/mL	8.0	(6.1–10.4)	6.1	(4.5–8.6)	n/a		< 0.001	–	–
BALP, U/L	21	(17–26)	28	(22–35)	n/a		< 0.001	–	–
(II) Longitudinal, women attending ALL three visits (*n* = 382*)*									
CTX, ng/L	258	(181–378)	292	(212–402)	257	(192–366)	< 0.001	0.33	0.001
PINP, ng/mL	48.8	(39.6–61.2)	49.8	(37.9–63.3)	44.0	(31.9–56.8)	0.49	< 0.001	< 0.001
TRAcP5b, U/L	3.2	(2.5–4.0)	5.0	(4.0–6.5)	3.8	(3.0–4.6)	< 0.001	< 0.001	< 0.001
U-OC, µg/mmol	1.0	(0.7–1.6)	1.3	(0.7–1.9)	2.1	(1.3–2.9)	0.005	< 0.001	< 0.001
tOC, ng/mL	8.1	(6.3–10.3)	6.0	(4.4–8.3)	n/a		< 0.001	–	–
BALP, U/L	21	(17–25)	28	(22–35)	n/a		< 0.001	–	–

Values are mean (SD), or number (%), for BTMs results are shown as medians (interquartile range, IQR), n/a, not available. *p values are paired samples *t* test (log-transformed values). The number of samples available for BTM analysis were: 999–1024 (baseline); 667–696 (5 years) and 342–369 (10 years)

### BTMs and Fracture Prediction in Women Age 75

**Fig. 2:**
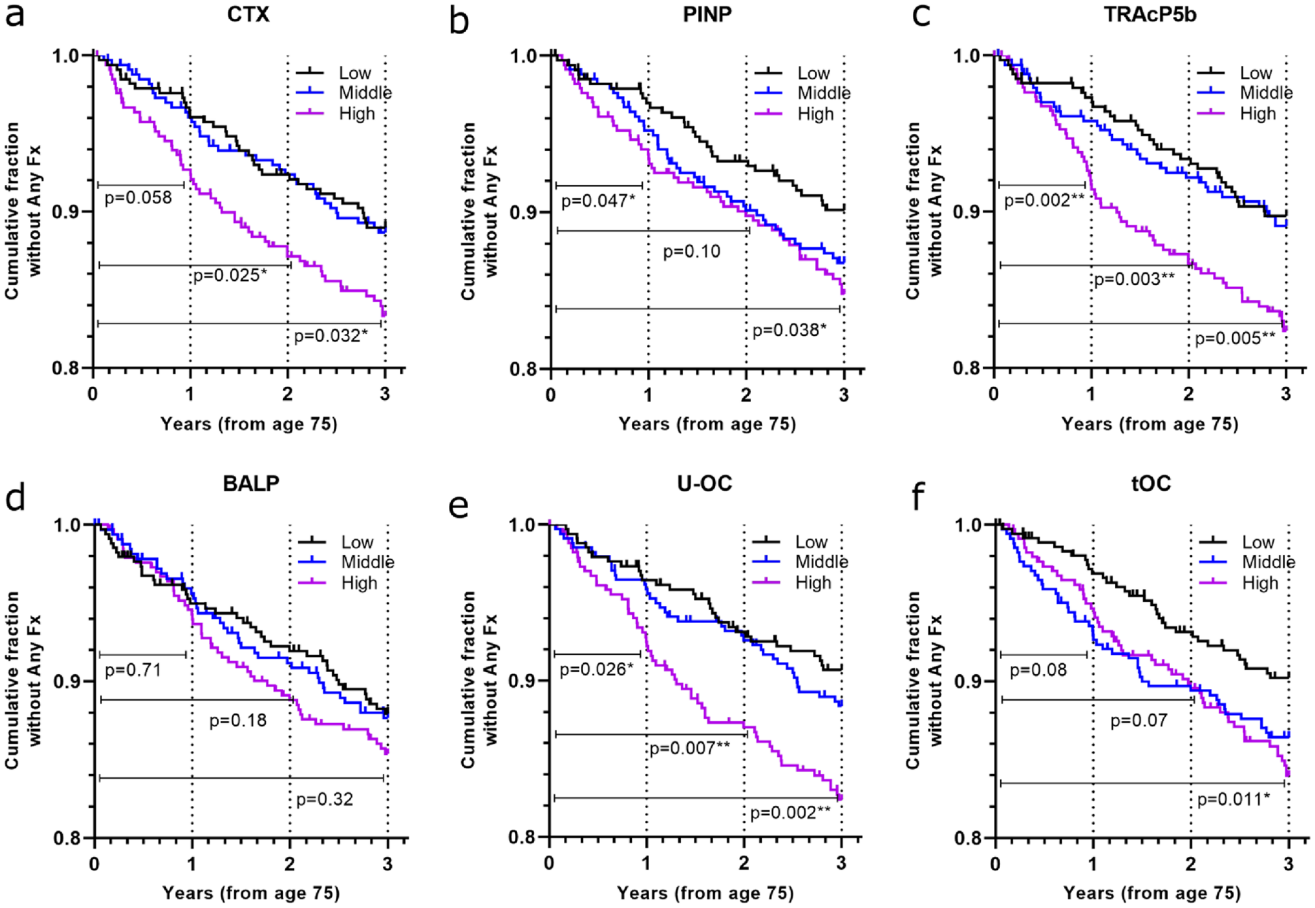
Short-term fracture risk for *any fracture* using BTMs measured at age 75. Cumulative fractions of women without *any fracture* are shown in a Kaplan–Meier curve for **a** CTX, **b** PINP, **c** TRAcP5b, **d** BALP **e** U-OC **f** tOC. Tertiles are Low (black), Middle (blue), and High (magenta). *P*-values (log rank test) for 1, 2 and 3 years are reported (*Tertiles Low vs. High,* unadjusted). End-point was date of first fracture (or end of follow-up time, if fracture free) and death-date as censor. Comparison was made with fracture-free women. The number of women with any fracture at the end of 1 year was 51, at 2 years (93), 3 years (130)

High CTX levels at age 75 were associated with increased risk of *any fracture* at 2 and 3 years after measurement (Table 2, Fig. 2a), with and without adjustment for smoking, bisphosphonate use and prior osteoporotic fracture (Table 2). The results were similar, or even more evident, with exclusion of bisphosphonate users (*n* = 33) (data not shown). With additional adjustment for BMD, the HRs for any fracture at 3 years were not significant (HR 1.52 (0.93–2.50), Table 2).

**Table 2:** SHORT-TERM fracture risk

	Fracture prediction over 1 year (i.e. from age 75 to 76)				Fracture prediction over 2 years (i.e. from age 75 to 77)				Fracture prediction over 3 years (i.e. from age 75 to 78)					
	Unadjusted		Adjusted*		Unadjusted		Adjusted*		Unadjusted		Adjusted*		Adjusted**	
Fracture Type	HR	95% CI	HR	95% CI	HR	95% CI	HR	95% CI	HR	95% CI	HR	95% CI	HR	95% CI
Any^a^	*n* = *51*				*n* = *93*				*n* = *130*					
CTX	1.90	(0.97–3.73)	1.78	(0.89–3.56)	**1.75**	**(1.07–2.88)**	**1.72**	**(1.03–2.88)**	**1.58**	**(1.04–2.42)**	**1.80**	**(1.14–2.83)**	1.52	(0.93–2.50)
PINP	2.11	(1.00–4.49)	2.09	(0.96–4.53)	1.57	(0.92–2.68)	1.62	(0.93–2.83)	**1.59**	**(1.02–2.48)**	**1.92**	**(1.19–3.09)**	1.59	(0.93–2.71)
TRAcP5b	**3.14**	**(1.48–6.64)**	**3.06**	**(1.43–6.55)**	**2.11**	**(1.27–3.51)**	**2.09**	**(1.24–3.51)**	**1.81**	**(1.18–2.75)**	**1.98**	**(1.28–3.07)**	1.51	(0.94–2.41)
U-OC	**2.15**	**(1.08–4.28)**	**2.05**	**(1.01–4.14)**	**1.97**	**(1.19–3.27)**	**1.96**	**(1.17–3.30)**	**1.99**	**(1.29–3.08)**	**2.22**	**(1.40–3.50)**	**1.84**	**(1.11–3.04)**
tOC	2.00	(0.90–4.45)	1.92	(0.85–4.31)	1.62	(0.95–2.75)	1.58	(0.92–2.73)	**1.75**	**(1.13–2.73)**	**1.94**	**(1.22–3.09)**	1.59	(0.97–2.62)
BALP	1.13	(0.59–2.18)	1.08	(0.56–2.10)	1.40	(0.85–2.30)	0.82	(0.50–1.35)	1.24	(0.81–1.88)	1.38	(0.89–2.14)	1.29	(0.79–2.10)
Major Osteoporotic^b^	*n* = *39*				*n* = *70*				*n* = *100*					
CTX	**2.28**	**(1.04–5.02)**	2.16	(0.95–4.91)	**2.03**	**(1.13–3.64)**	**1.97**	**(1.07–3.61)**	1.60	(0.99–2.58)	**1.77**	**(1.06–2.94)**	1.37	(0.78–2.39)
PINP	**2.44**	**(1.01–5.89)**	2.49	(0.99–6.23)	1.59	(0.85–2.98)	1.63	(0.85–3.11)	1.61	(0.97–2.65)	**1.87**	**(1.10–3.20)**	1.38	(0.77–2.48)
TRAcP5b	**2.55**	**(1.12–5.80)**	**2.51**	**(1.09–5.79)**	1.58	(0.90–2.78)	1.54	(0.87–2.73)	1.56	(0.98–2.51)	**1.66**	**(1.02–2.70)**	1.24	(0.73–2.09)
U-OC	1.80	(0.86–3.77)	1.68	(0.78–3.60)	**1.83**	**(1.03–3.27)**	1.76	(0.97–3.20)	**1.83**	**(1.13–2.97)**	**1.98**	**(1.19–3.30)**	1.53	(0.87–2.67)
tOC	1.64	(0.68–3.96)	1.57	(0.64–3.85)	1.62	(0.88–2.98)	1.56	(0.84–2.89)	**1.64**	**(1.00–2.68)**	**1.79**	**(1.07–2.99)**	1.47	(0.85–2.55)
BALP	1.25	(0.60–2.60)	1.20	(0.57–2.53)	1.51	(0.85–2.71)	1.50	(0.83–2.72)	1.41	(0.87–2.27)	1.55	(0.94–2.55)	1.37	(0.79–2.38)
Vertebral^c^	*n* = *15*				*n* = *22*				*n* = *37*					
CTX	2.57	(0.81–8.20)	2.18	(0.68–7.01)	1.67	(0.65–4.31)	1.35	(0.52–3.50)	1.25	(0.59–2.62)	1.13	(0.53–2.43)	0.67	(0.29–1.55)
PINP	2.70	(0.72–10.19)	2.46	(0.65–9.22)	1.70	(0.62–4.69)	1.54	(0.56–4.23)	2.06	(0.93–4.59)	2.07	(0.90–4.74)	1.33	(0.56–2.14)
TRAcP5b	2.77	(0.73–10.43)	2.49	(0.66–9.40)	1.37	(0.51–3-68)	1.24	(0.46–3.34)	1.71	(0.77–3.76)	1.68	(0.75–3.74)	1.31	(0.56–3.09)
U-OC	4.18	(0.89–19.67)	3.64	(0.76–17.32)	2.16	(0.74–6-32)	1.73	(0.58–5.14)	**2.72**	**(1.20–6.16)**	**2.46**	**(1.05–5.76)**	1.56	(0.64–2.78)
tOC	3.53	(0.73–16.97)	3.09	(0.64–14.19)	2.05	(0.70–4.69)	1.76	(0.60–5.16)	**2.69**	**(1.13–6.45)**	**2.63**	**(1.07–6.47)**	1.84	(0.72–4.67)
BALP	3.04	(0.82–11.23)	2.72	(0.74–10.06)	2.85	(0.91–8.96)	2.54	(0.81–7.97)	**4.05**	**(1.52–10.79)**	**4.27**	**(1.54–11.89)**	**3.41**	**(1.16–10.0)**

Ability of BTMs measured at age 75 to predict fractures over the time span of 1, 2 and 3 years. Values are hazard ratios (HR) with 95% confidence intervals (CI) for 1^st^ new fracture for women in the highest BTM tertile (Cox regression analysis), comparing women with fracture to fracture-free women (reference = Tertile_Low_). HRs with the lower limit of the 95% CI ≥ 1 are highlighted with bold text

^*^Adjusted for baseline smoking, bisphosphonate use and prior osteoporotic fracture (between ages 50–75)

^**^Adjusted for baseline smoking, bisphosphonate use and prior osteoporotic fracture (between ages 50–75) and baseline total hip aBMD tertile

^a^Any fracture (not including fractures of toes, finger, face, or resulting from pathology or high energy)

^b^Major osteoporotic fractures (as defined by FRAX, i.e. hip, vertebral, distal radius and proximal humerus)

^c^Vertebral fractures confirmed from x-rays

Women with high CTX levels had a higher cumulative percentage of fractures during the *five-year* period (75 year–80 year; *p* = 0.029) (Supplemental Fig. 2) and 5 year fracture risk of any fracture was elevated with or without adjustment (HR 1.45, HR_Adj_ 1.59) (Table 3). Over 10 years*,* the association attenuated (HR 1.28, HR_Adj_ 1.37), although women with high levels still tended to sustain more fractures. There was no association at the end of the 15 year follow up. With regards to specific fracture types, high CTX levels were consistently associated with risk of major osteoporotic fractures (MOF), both short term and up to 10 years (Tables 2 and 3)*.* As shown in *Supplemental *Fig. 4, one SD increase in CTX was associated with increased MOF risk, particularly at 2–5 years. Vertebral fractures were not predicted by CTX, nor were hip fractures (data not shown).

**Table 3:** LONG-TERM fracture risk

	Fracture prediction over 5 years (i.e. from 75 to 80)				Fracture prediction over 10 years (i.e. from 75 to 85)				Fracture prediction over 15 years (i.e. from 75 to 90)			
	Unadjusted		Adjusted*		Unadjusted		Adjusted*		Unadjusted		Adjusted*	
Fracture type	HR	95% CI	HR	95% CI	HR	95% CI	HR	95% CI	HR	95% CI	HR	95% CI
Any^a^	*n* = *214*				*n* = *399*				*n* = *524*			
CTX	**1.45**	**(1.04–2.03)**	**1.59**	**(1.11–2.26)**	**1.28**	**(1.00–1.64)**	**1.37**	**(1.06–1.77)**	1.17	(0.95–1.45)	1.24	(0.99–1.56)
PINP	1.21	(0.86–1.70)	1.34	(0.94–1.90)	1.04	(0.81–1.34)	1.13	(0.87–1.46)	0.92	(0.45–1.14)	0.99	(0.80–1.24)
TRAcP5b	1.36	(0.98–1.89)	**1.44**	**(1.03–2.02)**	1.14	(0.90–1.46)	1.20	(0.94–1.54)	1.02	(0.83–1.26)	1.07	(0.87–1.33)
U-OC	**1.49**	**(1.07–2.08)**	**1.55**	**(1.10–2.19)**	1.24	(0.98–1.58)	1.28	(0.99–1.64)	1.12	(0.91–1.39)	1.14	(0.92–1.42)
tOC	1.26	(0.90–1.77)	1.34	(0.94–1.90)	1.05	(0.83–1.35)	1.11	(0.86–1.43)	0.94	(0.76–1.16)	0.99	(0.80–1.23)
BALP	1.13	(0.81–1.56)	1.21	(0.86–1.69)	1.04	(0.82–1.33)	1.10	(0.86–1.41)	0.99	(0.80–1.22)	1.05	(0.85–1.30)
Major osteoporotic^b^	*n* = *171*				*n* = *349*				*n* = *453*			
CTX	**1.60**	**(1.10–2.34)**	**1.75**	**(1.17–2.61)**	**1.33**	**(1.02–1.73)**	**1.42**	**(1.07–1.88)**	1.21	(0.96–1.52)	**1.29**	**(1.01–1.64)**
PINP	1.25	(0.86–1.82)	1.37	(0.92–2.03)	1.00	(0.77–1.31)	1.08	(0.82–1.42)	0.90	(0.71–1.13)	0.98	(0.77–1.24)
TRAcP5b	1.24	(0.87–1.79)	1.31	(0.90–1.89)	1.05	(0.81–1.36)	1.10	(0.84–1.43)	0.97	(0.77–1.21)	1.02	(0.81–1.29)
U-OC	**1.51**	**(1.04–2.17)**	**1.53**	**(1.05–2.25)**	1.23	(0.95–1.59)	1.25	(0.96–1.64)	1.13	(0.90–1.41)	1.14	(0.90–1.44)
tOC	0.99	(0.68–1.43)	1.25	(0.85–1.85)	0.91	(0.71–1.19)	1.04	(0.80–1.36)	0.84	(0.67–1.06)	0.94	(0.74–1.19)
BALP	1.29	(0.90–1.85)	1.39	(0.95–2.01)	1.09	(0.85–1.42)	1.16	(0.89–1.51)	1.03	(0.82–1.29)	1.10	(0.87–1.39)
Vertebral^c^	*n* = *65*				*n* = *152*				*n* = *214*			
CTX	1.21	(0.68–2.12)	1.16	(0.64–2.09)	1.20	(0.80–1.79)	1.27	(0.83–1.95)	1.03	(0.74–1.44)	1.09	(0.77–1.54)
PINP	1.23	(0.69–2.20)	1.31	(0.71–2.39)	0.90	(0.67–1.34)	1.00	(0.67–1.52)	0.77	(0.56–1.07)	0.86	(0.61–1.21)
TRAcP5b	1.48	(0.83–2.63)	1.52	(0.85–2.74)	1.03	(0.71–1.51)	1.12	(0.76–1.66)	0.93	(0.67–1.29)	1.03	(0.73–1.45)
U-OC	**1.99**	**(1.10–3.60)**	1.78	(0.96–3.30)	1.10	(0.75–1.60)	1.04	(0.69–1.55)	0.97	(0.70–1.34)	0.93	(0.66–1.31)
tOC	1.27	(0.72–2.21)	1.61	(0.87–2.99)	0.92	(0.63–1.35)	1.11	(0.74–1.67)	0.77	(0.56–1.07)	0.89	(0.63–1.25)
BALP	1.78	(0.99–3.18)	**1.91**	**(1.04–3.49)**	1.08	(0.74–1.58)	1.18	(0.80–1.76)	0.95	(0.69–1.31)	1.04	(0.74–1.44)

Ability of BTMs measured at age 75 to predict fracture over a time span of 5, 10, 15 years. Values are hazard ratios (HR) with 95% confidence intervals (CI) for 1^st^ new fracture for women in the highest BTM tertile (Cox regression analysis), comparing women with fracture to fracture-free women (reference = Tertile_Low_). HRs with the lower limit of the 95% CI ≥ 1 are highlighted with bold text

^*^Adjusted for baseline smoking, bisphosphonate use and prior osteoporotic fracture (between ages 50–75)

^a^Any fracture (not including fractures of toes, finger, face, or resulting from pathology or high energy)

^b^Major osteoporotic fractures (as defined by FRAX, i.e. hip, vertebral, distal radius and proximal humerus)

^c^Vertebral fractures confirmed from x-rays

PINP was not associated with 1 or 2 years risk, but high levels were associated with higher 3 year risk of any fracture (Table 2); shown also in the Kaplan-Meir analysis (Fig. 2b) (Tertile_High_ vs Tertile_Low_, *p* = 0.038 at 3 years). Results were similar with adjustment for smoking, bisphosphonate use and prior osteoporotic fracture, but with additional adjustment for BMD (baseline total hip) the HRs for any fracture at 3 years lost significance (HR 1.59 (0.93–2.71), *Supplemental *Table 2). PINP was not associated with long term fracture risk (5–15 years). With regards to specific fracture types, PINP may predict MOF over 1 year, but not other fracture types or over longer time-frames (Tables 2 and 3)*.*

High levels of TRAcP5b were consistently associated with increased risk of any fracture at 1, 2 and 3-years after measurement with and without adjustment (Table 2, Fig. 2c) and with exclusion of bisphosphonate users (*N* = 33) (data not shown). Beyond this time period, however, the cumulative percentage of fractures during the five-year period (75 year–80 year) did not differ between tertiles of TRAcP5b (*Supplemental *Fig. 2) and long term fracture risk was not associated with TRAcP5b (Table 2).

BALP was not associated with short-term or long term fracture risk (Table 2, Table 3, Fig. 2d).

High levels of U-OC were consistently associated with increased risk of any fracture already within one year and at 2 and 3 years after measurement, with and without adjustment for smoking, bisphosphonate use and prior osteoporotic fracture (Table 2, Fig. 2e). As with the other BTMs, exclusion of bisphosphonate users tended to strengthen the results (data not shown). The cumulative percentage of fractures during the five-year period (75 year–80 year) was higher for women with high levels of U-OC (*p* = 0.018) (*Supplemental *Fig. 2) and Cox regression analysis showed an elevated 5 year fracture risk (HR 1.49, 1.07–2.08) (Table 3). As above, the results are similar with and without adjustment. Over ten and 15 years, the association with U-OC was attenuated and although women with high levels tended to sustain more fractures there was no associated risk. Interestingly, U-OC was consistently associated with MOF and even vertebral fracture over 3 and 5 years (Tables 2 and 3), although not hip fracture (data not shown).

High levels of tOC were associated with higher 3 year fracture risk, with or without adjustment (Table 2). Kaplan–Meier analysis also showed this higher risk for any fracture (Tertile_High_ vs Tertile_Low,_ *p* = 0.011) (*Fig. **2**f*). tOC was not associated with long term fracture risk (5–15 years) (Table 3).

### BTMs and Fracture Prediction in Women Age 80

Neither CTX, PINP nor the four other BTMs when measured at age 80 were associated with fracture risk, in any of the time-frames (1 to 3 year, or 5 year) (*Supplemental *Table 3*; Supplemental *Fig. 3 ). Even a combination of two consecutive measurements of the same BTM (at 75 and 80) did not enhance overall fracture prediction beyond age 80 (*data not shown)*.

**Fig. 3:**
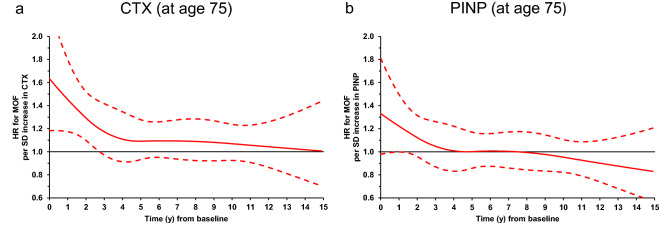
Hazard ratios (HR) for *major osteoporotic fracture (MOF)*. To model the predictive value of BTMs over time, MOF risk (HR per SD change in BTMs) was analyzed using a modified Poisson regression model for **a** CTX and **b** PINP measured at age 75 adjusted for baseline smoking, bisphosphonate use, prior osteoporotic fracture. Solid lines represent HRs; dotted lines the 95% CIs

Modelling the predictive value of bone turnover markers as a function of time using Poisson regression and HR per SD increase in BTM Z-score, we can illustrate the utility of BTMs over time and with age. Measured at age 75 CTX was predictive for MOFs up to 2.3 years (HR 1.19, 1.00–1.41) or 2.7 years after adjustment (HR_Adj_ 1.20, 1.00–1.44) (*Fig. **3**a*). Measured at age 80, CTX was no longer predictive for MOFs in any time perspective. For PINP measured at age 75, predictive value may be as little as 1 year (HR 1.17 (0.95–1.44); HR_Adj_ 1.22 (1.00–1.50) (*Fig. **3**b*) and does not appear to be useful at older ages.

## Discussion

In this large study of one thousand elderly women, bone turnover was profiled over ten years using six bone turnover markers capturing different aspects of bone metabolism. The study demonstrated the longitudinal change in BTM values during ageing and confirmed the ability of BTMs to predict fracture risk, particularly in the short-term. The ability to predict fracture attenuated over time and age. In concordance with the recent meta-analysis [5], CTX appears to be the most robust marker for future fracture risk.

Measured at age 75, high levels of CTX were consistently associated with fracture risk, including major osteoporotic fractures, across all time-frames both short and long. Although there is some attenuation of the HR’s over time, CTX appears to be a robust marker for fracture prediction, albeit not specifically for hip or vertebral fractures. This inability to discriminate is consistent with existing data [33–35], but could also be explained by an insufficient number of hip fractures, or that vertebral fractures were not clinically defined. While attempting to capture as many vertebral fractures as possible we can presume this to be an underestimate [36]. Particularly for hip fractures it is likely that factors such as predisposition to fall or general frailty attenuates the importance of the instantaneous bone metabolic status. In contrast, PINP was not consistently associated with fracture risk over time in this cohort of aging women, which could reflect a generally reduced bone formation rate at this stage in the life-course. This finding suggests that it may be more relevant to use PINP when investigating osteoporosis in younger patients.

For other bone turnover markers, although directionally in concordance while not consistently statistically significant, resorption markers were clearly more commonly associated with increased fracture risk, particularly in the short term; with the exception of BALP. For example, TRAcP5b appears to be robust in predicting any fracture within a three year time frame. Of interest is also that high levels of urinary osteocalcin were consistently associated with elevated short-term fracture risk. This included major osteoporotic fractures, but also vertebral. While replication in other studies is necessary, this speaks for its potential value, given the difficulty to identify vertebral fractures which are often asymptomatic, while degenerative changes, common in the elderly, can misleadingly indicate higher BMD [37].

We did not systematically adjust for BMD, since it is undoubtedly a stronger predictor of fracture risk than bone turnover markers [38] and has been shown previously in this cohort [6, 7]. In the sensitivity analyses, fracture risk was, as expected, attenuated. However, even if bone turnover markers are not independent of BMD, this does not necessarily undermine their value. On the contrary it provides an instrument capturing underlying metabolic aspects of overall skeletal health. While low BMD at a given assessment captures the end-point of earlier bone loss, high BTMs indicate high global skeletal turnover that may predispose to future bone loss and deterioration of bone architecture or quality, particularly the trabecular network prominent in vertebrae. Being more dynamic than BMD, bone turnover markers are routinely employed for rapid monitoring of both anti-resorptive and anabolic treatment [39] and our analyses indicate that fracture incidence in those with high or low marker levels begins to differ already within the first twelve months of measurement. This suggests that the ‘snap-shot’ of bone turnover reflected by BTM assessment provides valuable evidence of skeletal health, which has clinical value for patient management.

Like most biomarkers there is attenuation with age, and here the association between bone turnover markers and fracture obviously attenuates [7] with our data showing that by age 80, BTM assessment is most likely not useful for fracture prediction. Furthermore, fracture risk prediction by bone markers does not extend over longer time-frames. For predicting long term fracture risk, bone turnover measured at a given time point appears to be less important than other accumulated risk factors, in elderly women well beyond menopause. Given this attenuation with age and over time, incorporation of BTMs, with the possible exception of CTX, into FRAX would be unlikely to benefit 10 year risk assessment. However, biomarkers might be usefully applied at transition stages in health during aging. The results from this study suggest that the ‘snap-shot’ of bone turnover reflected by BTM assessment does in fact provide valuable information of current skeletal health and could provide additional information as an adjuvant tool with other risk assessment methods. In the very old, low bone mass, propensity to fall and composite measures of health are more relevant indicators of fracture risk [40–42].

The major strengths of this study include, firstly the clinically relevant cohort of women at demographically high fracture risk, large sample size and homogeneity. This includes the single age of all participants, which helps minimize variation in marker levels associated with chronological age, even if biological age differs. The six bone turnover markers capture all aspects of bone turnover, and measured at three time-points over 10 years, capture the remaining-lifetime perspective of elderly women. Recommended reference markers, CTX and PINP, were assessed at all time-points and in conjunction, we had confirmed fracture data for up to 15 years post-baseline. We envisage these data are appropriate to include in future meta-analyses to estimate effect sizes of BTMs for fracture prediction.

Secondly, we used Z-scores (rather than concentrations) when analyzing HR per SD change in markers which minimizes fluctuations in BTM measures over time (due to methodological changes and sample storage). It also provides evidence that the association between fracture risk and (some or all) bone turnover markers is not necessarily linear, rather that BTMs must be above a threshold before they are associated with fracture risk. By also analysing BTM tertiles we could more clearly demonstrate the ability of bone markers to predict short-term risk of osteoporotic fracture, which would have otherwise been missed. For the majority, the same tertile is maintained across sampling times [43].

Z-scores do not provide generalized diagnostic thresholds for bone turnover markers. While thresholds and ROC analysis have been used in intervention studies to identify responders and non-responders to anti-resorptive therapies [8, 39], employing specific cut-points for bone turnover may be sub-optimal in a trauma-dependent fracture prediction setting. Finally, prior fracture is known to influence bone turnover markers levels, therefore this was included in the adjusted models (along with smoking and bisphosphonate use) and did not alter the results.

As with all observational studies, the results should be interpreted cautiously and we acknowledge the limitations of this study. First are the collection of serum in non-fasted state and single measurement. However, we show that CTX (the BTM most affected by feeding) is very highly correlated in non-fasted and fasted samples and that the majority of women are similarly classified in tertiles from both samples. Overall, this demonstrates that non-fasted/fasted status per se is less relevant; most important is consistency in sampling. We have previously shown that serial assessment of turnover (four samplings over 5 years), is more strongly associated to bone loss than a single BTM measurement [43]. We also showed that the prediction of 9 year fracture risk was more consistent using the average of two resorption marker measurements taken within one year [7]. Second**,** a confounding effect on BTMs from fractures sustained during the study, is possible, since BTMs increase after fracture [9, 44], remaining higher than pre-fracture levels up to a year or beyond [45]. However, adjustment for fractures sustained *prior to baseline* led to fracture risk being generally more pronounced. Even adjusting for fracture within the previous 2 years (between ages 73–75) instead of prior osteoporotic fracture (between ages 50–75) had minimal effect on 1, 2 or 3 year HRs (data not shown). Furthermore, in a sub-analysis using CTX, the risk of any fracture over 1, 2 and 3 years was elevated in women without a fracture prior to baseline, although the confidence intervals were wide due to low number (data not shown). Therefore, while recognizing that a previous fracture is one of the strongest risk factors for subsequent fractures [36], our data suggests that even so, markers can pick up a risk signal in the elderly.

Third, while bone active medications may influence marker profiles, in the OPRA cohort their use was rare (3%, 7%, 12% at respective visits) since the study started in the late 1990’s when use of bone active medications was relatively low. Warfarin use was also rare (6% at 5 year; 11% at 10 year). Fourth, inherent in the longitudinal investigation of an already elderly cohort, there is an increase in comorbidities and medications as well as fewer attending follow-up, although cause of mortality is unknown. To address the loss to follow-up, we also reported on those women attending all three visits and also analyzed CTX and PINP using death as a competing risk, with little difference in HRs (Supplemental Table 2). Being cautious, it is possible that different trajectories of bone loss may influence the predictive ability of bone turnover markers at age 75, while conversely by age 80 there may be a selection bias towards healthier individuals who were able to participate; alternately, it reflects reduced power with follow-up. Finally, we acknowledge that multiple tests have been performed, although the confidence intervals, in conjunction with consistent direction of BTM effect, suggests biological plausibility. We cannot fully explain the attenuation of association with age, although sample size is one possibility; other factors may include fall propensity, comorbidity and frailty. The results should be interpreted with these in mind and cannot be extrapolated to younger women, elderly men or other ethnic groups.

In conclusion, the principal contribution from this comprehensive analysis of bone markers in older women, is the demonstration of their ability to inform on fracture risk in a short term, one to three year, perspective, whereas notably, in the long-term or above age 80 years, bone markers appear less valuable. Bone resorption markers, particularly CTX, were more consistently associated with fracture risk than formation markers in the very elderly.

## Supplementary Information

Below is the link to the electronic supplementary material.

Supplementary file1 (DOCX 1114 KB)

## Data Availability

Data are available upon request.
