# Supplementary material for: Bone Turnover Marker Profiling and Fracture Risk in Older Women: Fracture Risk from Age 75 to 90
Source: Calcif Tissue Int. 2022 Jun 24;111(3):288–99. doi: 10.1007/s00223-022-00996-8 (PMC9395308; doi:10.1007/s00223-022-00996-8)
Supplement: Supplementary file 1 — Supplementary file1 (DOCX 1114 KB) [file 223_2022_996_MOESM1_ESM.docx]

**Bone Turnover Marker Profiling and Fracture Risk in Older Women: Fracture Risk from Age 75 to 90**

Kaisa K. Ivaska^1,2^, Fiona E. McGuigan^2^, Linnea Malmgren^2, 3^, Paul Gerdhem^2,4,5^, Helena Johansson^6, 7^, John A. Kanis^6, 7^, Kristina E. Akesson^2,8^

^1^Institute of Biomedicine, University of Turku, Turku, Finland; ^2^Clinical and Molecular Osteoporosis Research Unit, Department of Clinical Sciences Malmö, Lund University, Malmö, Sweden; ^3^Department of Geriatrics, Skåne university hospital, Malmö, Sweden; ^4^Department of Clinical Science, Intervention and Technology, Karolinska Institutet, Stockholm. Sweden; ^5^Department of Surgical Sciences, Uppsala University and Department of Orthopaedics, Uppsala University Hospital, Uppsala, Sweden; ^6^Centre for Metabolic Bone Diseases, University of Sheffield, Sheffield, UK; ^7^Mary McKillop Institute for Health Research, Australian Catholic University, Melbourne, Australia; ^8^Department of Orthopaedics, Skåne University Hospital, Malmö, Sweden

**Running headline**: Bone turnover marker profiling and fracture

**Correspondence:**

Kristina Åkesson (Orcid 0000-0003-3024-2804)

Department of Orthopedics Malmö, Skåne University Hospital, S-214 28 Malmö, Sweden

Email: [kristina.akesson@med.lu.se](mailto:kristina.akesson@med.lu.se)

Kaisa Ivaska (Orcid 0000-0001-7482-7623)

University of Turku, Finland

Email: [kaisa.ivaska@utu.fi](mailto:kaisa.ivaska@utu.fi)

**Supplemental Table 1.** Number of WOMEN with fractures (I) and number of FRACTURES (II) in the cohort within each time period and cumulatively

|  | **I**  **Number Of Women With Fractures*** | | | | | | **II**  **Number Of Fractures** ^#^ | | | |
| --- | --- | --- | --- | --- | --- | --- | --- | --- | --- | --- |
| Fracture Type | **Cumulative**  **No. of women**  with at least one fracture  by 5 years | | **Cumulative**  **No. of women**  with at least one fracture  by 10 years | | **Cumulative**  **No. of women**  with at least one  fracture  by end of study | | **Total No.**  **between age 75-80** | **Total No.**  **between age 80-85** | **Total No**.  **between age 85-90** | **Cumulative**  **No. of Fractures**  at end of  follow-up |
|  |  |  |  |  |  |  |  |  |  |  |
| Any^1^ | 214 | 20.5 % | 399 | 38.2 % | 524 | 50.2 % | 298 | 403 | 347 | 1048 |
| Major Osteoporotic^2^ | 171 | 16.4 % | 349 | 33.4 % | 453 | 43.4 % | 230 | 334 | 251 | 815 |
|  |  |  |  |  |  |  |  |  |  |  |
| Hip | 48 | 4.6 % | 130 | 12.5 % | 195 | 18.7 % | 50 | 101 | 79 | 230 |
| Vertebral^3^ | 65 | 6.2 % | 152 | 14.6 % | 214 | 20.5 % | 98 | 136 | 97 | 331 |
| Radius | 53 | 5.1 % | 100 | 9.6 % | 138 | 13.2 % | 53 | 53 | 43 | 149 |
| Shoulder | 28 | 2.7 % | 70 | 6.7 % | 100 | 9.6 % | 29 | 44 | 32 | 105 |
| Pelvic | 21 | 2.1 % | 42 | 4.0 % | 71 | 6.8 % | 21 | 23 | 33 | 77 |
| Other^4^ | 44 | 4.2 % | 81 | 7.8 % | 132 | 12.6 % | 47 | 46 | 63 | 156 |
|  |  |  |  |  |  |  |  |  |  |  |
| No. Alive (%) | 914 | 90.1 % | 737 | 70.6 % | 446 | 42.7 % |  |  |  |  |

** First fracture was used as the endpoint. A woman may be included in multiple categories if different fracture types were sustained during follow-up.*

*^#^ Multiple fractures in the same woman are included in the count.*

*^1^Any fracture (not including toe,s fingers or face, pathological fractures or fractures resulting from traffic accidents)*

*^2^Major osteoporotic fractures (as defined in the FRAX algorithm i.e. hip, vertebral, distal radius, shoulder)*

*^3^Vertebral fractures confirmed from x-rays*

*^4^Other sites include tibia, fibula, ulna, clavicle and ribs (and are included in any fractures^1^)*

**Supplemental Table 2. Ability of BTMs measured at age 75 to predict any fracture over three years – the effect of competing risk of death.** Hazard ratios (HR) with death as a competing risk (Fine and Gray competing risk regression model) are provided.

| **Fine & Gray**  **regression model** | **Fracture prediction over 3 years**  ***(i.e. from 75 to 78)*** | | | |
| --- | --- | --- | --- | --- |
|  | Unadjusted | | *Adjusted | |
|  | HR | 95% CI | HR | 95% CI |
| **Any fracture** |  |  |  |  |
| CTX | **1.56** | **(1.03-2.38)** | **1.77** | **(1.12-2.80)** |
| PINP | **1.61** | **(1.03-2.50)** | **1.94** | **(1.21-3.12)** |

*HRs are for 1^st^ new any fracture for the highest tertile (Tertile_High_) with 95% CI’s, comparing women with fracture (n=130) to fracture-free women (reference, Tertile_Low_).* **Adjusted for baseline smoking, bisphosphonate use and prior osteoporotic fracture (between ages 50-75).*

**Supplemental Table 3. The ability of BTMs to predict fracture risk is poor at age 80.** BTMs were measured at age 80 and ability to predict fractures over durations of 1-5 years was assessed.

|  | **Fracture prediction over 1 y**  ***(i.e. from 80 to 81)*** | | **Fracture prediction over 2 y**  ***(i.e. from 80 to 82)*** | | **Fracture prediction over 3 y**  ***(i.e. from 80 to 83)*** | | **Fracture prediction over 5 y**  ***(i.e. from 80 to 85)*** | |
| --- | --- | --- | --- | --- | --- | --- | --- | --- |
|  | Unadjusted | | Unadjusted | | Unadjusted | | Unadjusted | |
|  | HR | 95% CI | HR | 95% CI | HR | 95% CI | HR | 95% CI |
| **Any Fracture** | *n=34* |  | *n=76* |  | *n=107* |  | *n=130* |  |
| CTX | 0.91 | (0.37-2.33) | 1.58 | (0.89-2.83) | 1.11 | (0.69-1.77) | 1.15 | (0.80-1.66) |
| PINP | 1.10 | (0.47-2.58) | 1.38 | (0.82-2.34) | 0.95 | (0.61-1.49) | 0.92 | (0.65-1.31) |
|  |  |  |  |  |  |  |  |  |
| TRAcP5b | 0.78 | (0.36-1.73) | 1.23 | (0.72-2.09) | 1.12 | (0.71-1.77) | 0.94 | (0.66-1.34) |
| U-OC | 0.93 | (0.42-2.03) | 1.39 | (0.80-2.40) | 1.05 | (0.67-1.64) | 0.99 | (0.69-1.43) |
| tOC | 1.18 | (0.53-2.64) | 1.13 | (0.66-1.94) | 0.80 | (0.50-1.28) | 0.93 | (0.64-1.33) |
| BALP | 1.61 | (0.66-3.92) | 1.19 | (0.66-1.12) | 0.88 | (0.54-1.44) | 0.95 | (0.65-1.39) |
|  |  |  |  |  |  |  |  |  |

*Values are unadjusted hazard ratios (HR) for the 1^st^ new any fracture for the highest tertile (Tertile_High_)* *with 95% CI, estimated by Cox regression analysis comparing women with any fracture to fracture-free women (reference, Tertile_low_).*

**Supplemental Figure 1**

*
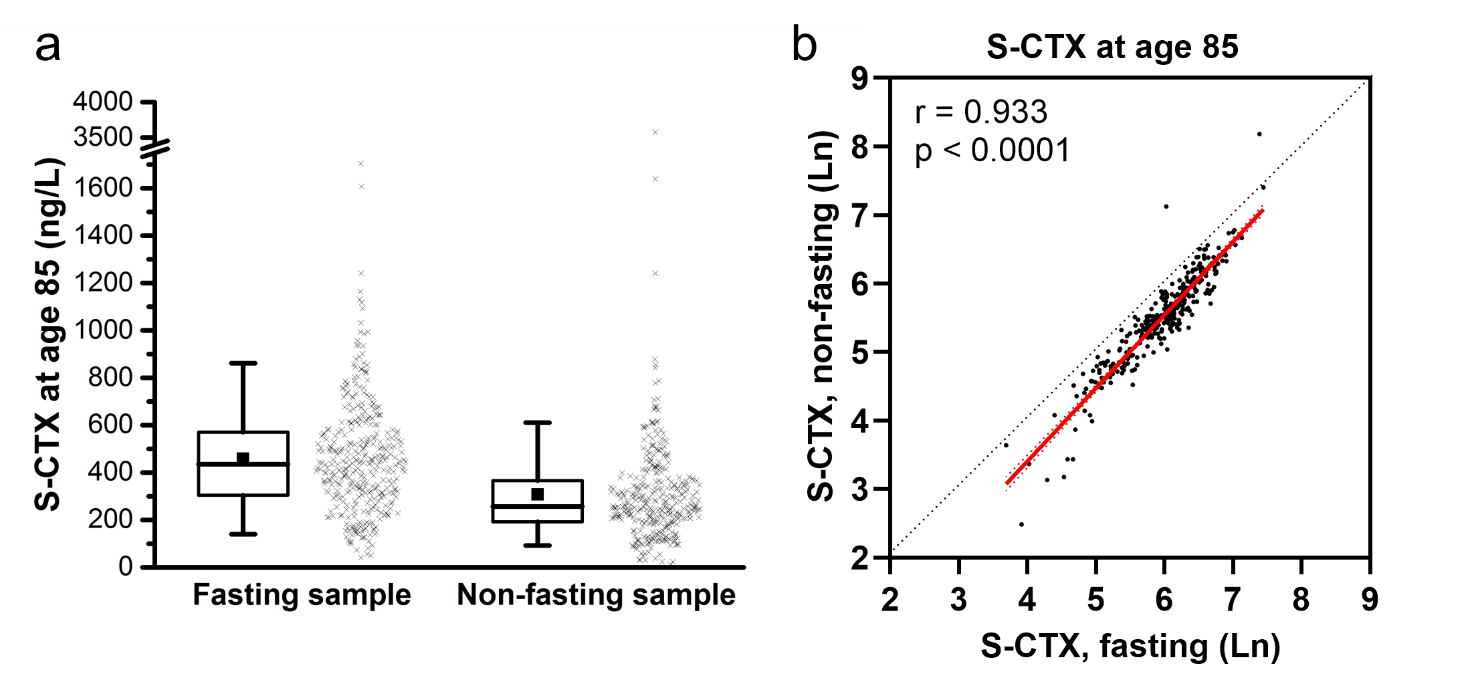
*

**Supplemental Figure 1.** a) CTX at age 85 measured in fasting samples and non-fasting samples. b) Correlation between fasting and non-fasting CTX values (both samples available N=336). Two serum samples were collected on the same day. Fasting samples were drawn around 8 am (N=365), thereafter a light breakfast were served and non-fasting samples were drawn, approximately 2 hours later (N=347). Serum CTX was analyzed in both non-fasting and fasting samples.

**Supplemental Figure 2**

**
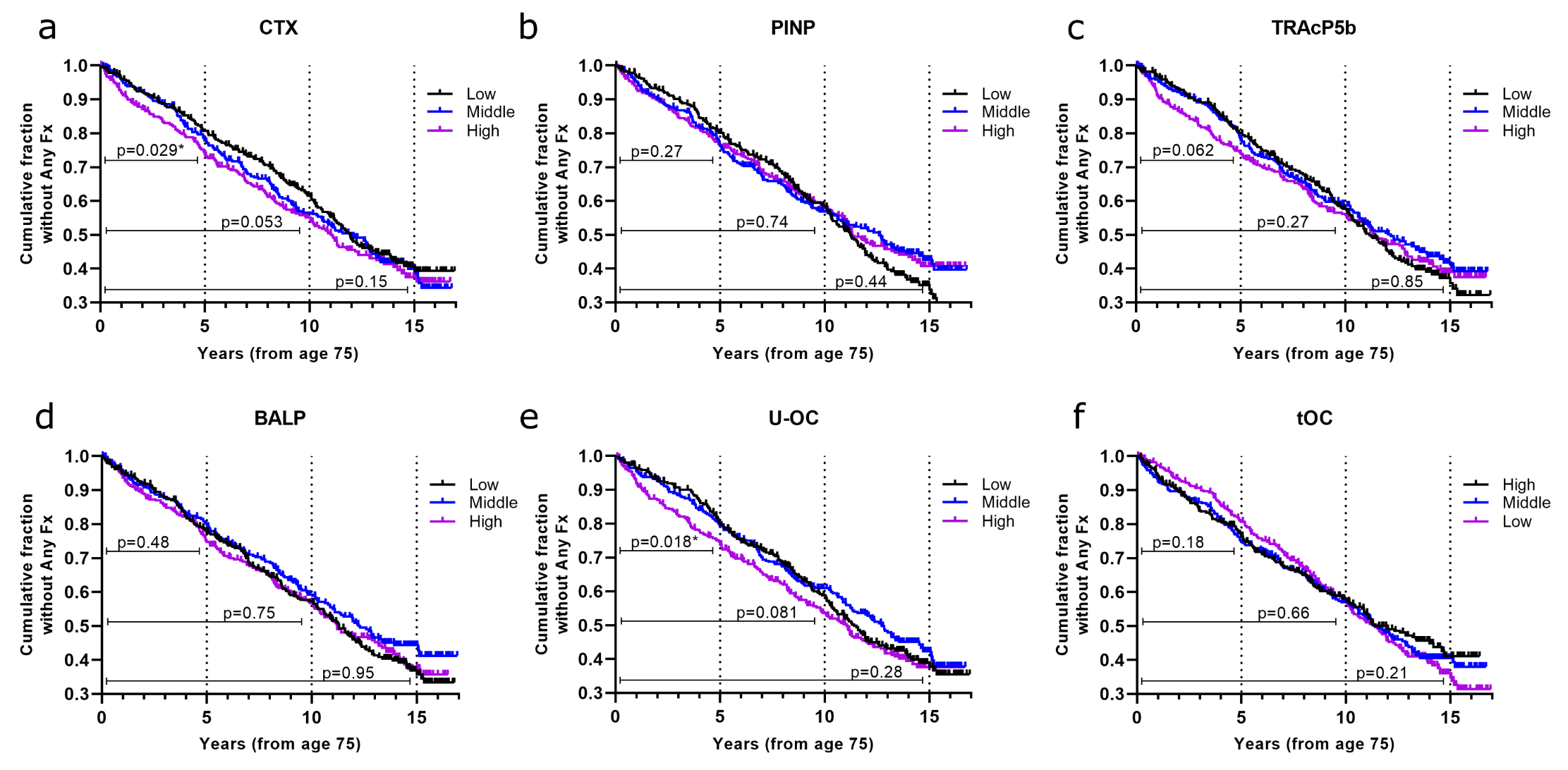
**

**Supplemental Figure 2. Long term fracture risk for *any fracture* using baseline BTMs (age 75).** Cumulative fractions of women without *any fracture* for tertiles of BTMs measured at baseline (age 75). Low (black), middle (blue), and high (magenta) tertiles of a) CTX, b) PINP, c) TRAcP5b, d) BALP, e) U-OC, and f) tOC are shown in a Kaplan-Meier curve. P-values for log rank test *(Tertile_Low_ vs. Tertile_High_*, unadjusted) for 5, 10 and 15-year follow-ups are reported. Number of women with fracture at the end of 5-years (214), 10-years (399) and 15-years (524). Comparison was made with fracture-free women with ‘time to first any fracture’ as endpoint.

**Supplemental Figure 3**

**
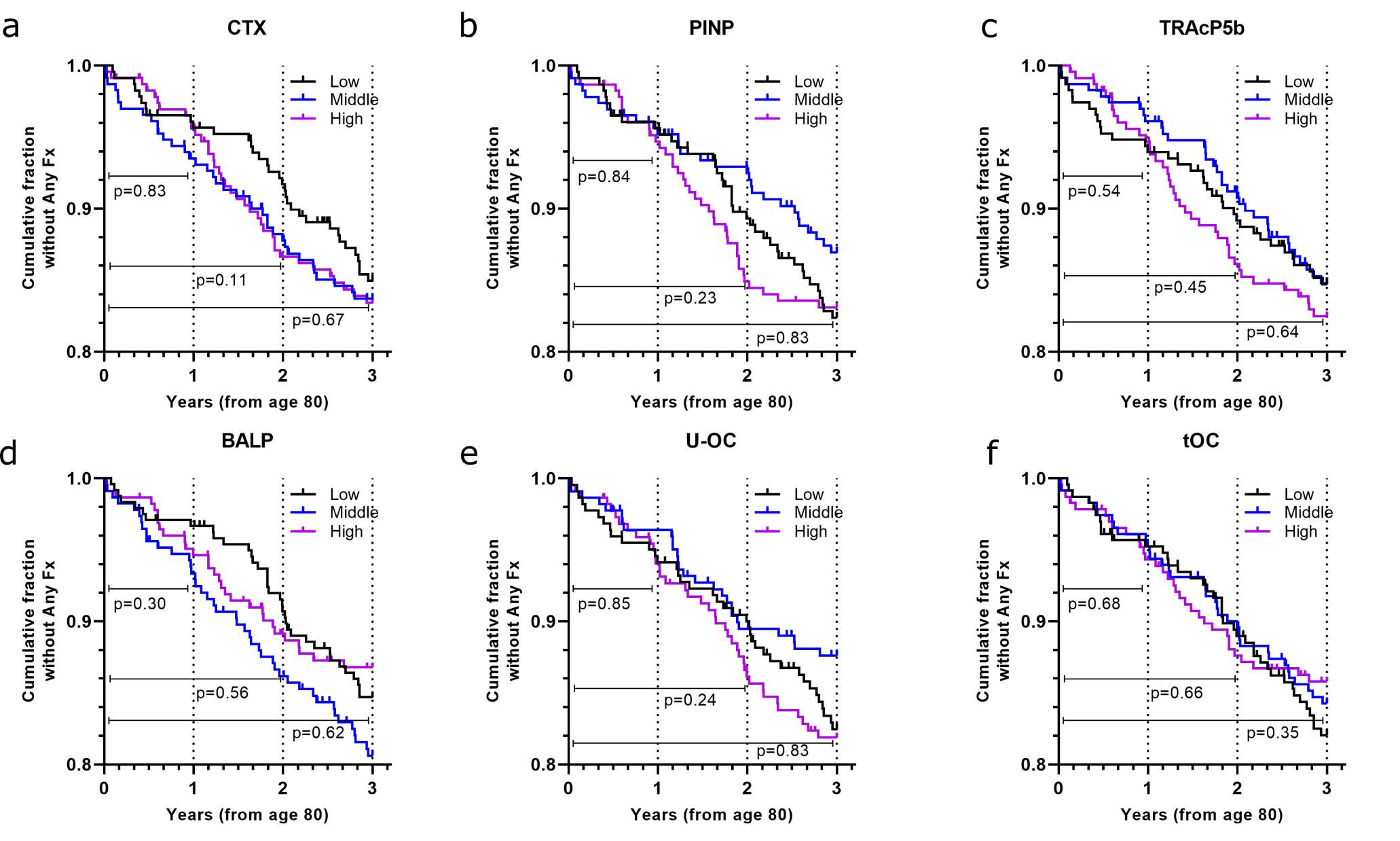
**

**Supplemental Figure 3. Short-term fracture risk for *any fracture* using BTMs at age 80 (Kaplan-Meier survival analysis).** Cumulative fractions of women without *any fracture* for tertiles of BTMs measured at baseline (age 75). Low (black), middle (blue), and high (magenta) tertiles of a) CTX, b) PINP, c) TRAcP5b, d) BALP, e) U-OC, and f) tOC are shown in a Kaplan-Meier curve. P-values for log rank test *(Tertile_Low_ (black) vs. Tertile_High_*, *(magenta)* unadjusted) for 1, 2 and 3 year follow-ups are reported. Nnumber of women with a fracture at the end of 1-year (68), 2-years (123) and 3-years (164). Comparison was made with fracture-free women with ‘time to first any fracture’ as endpoint. Note the scale in y-axis.

**Supplemental Figure 4.**

**
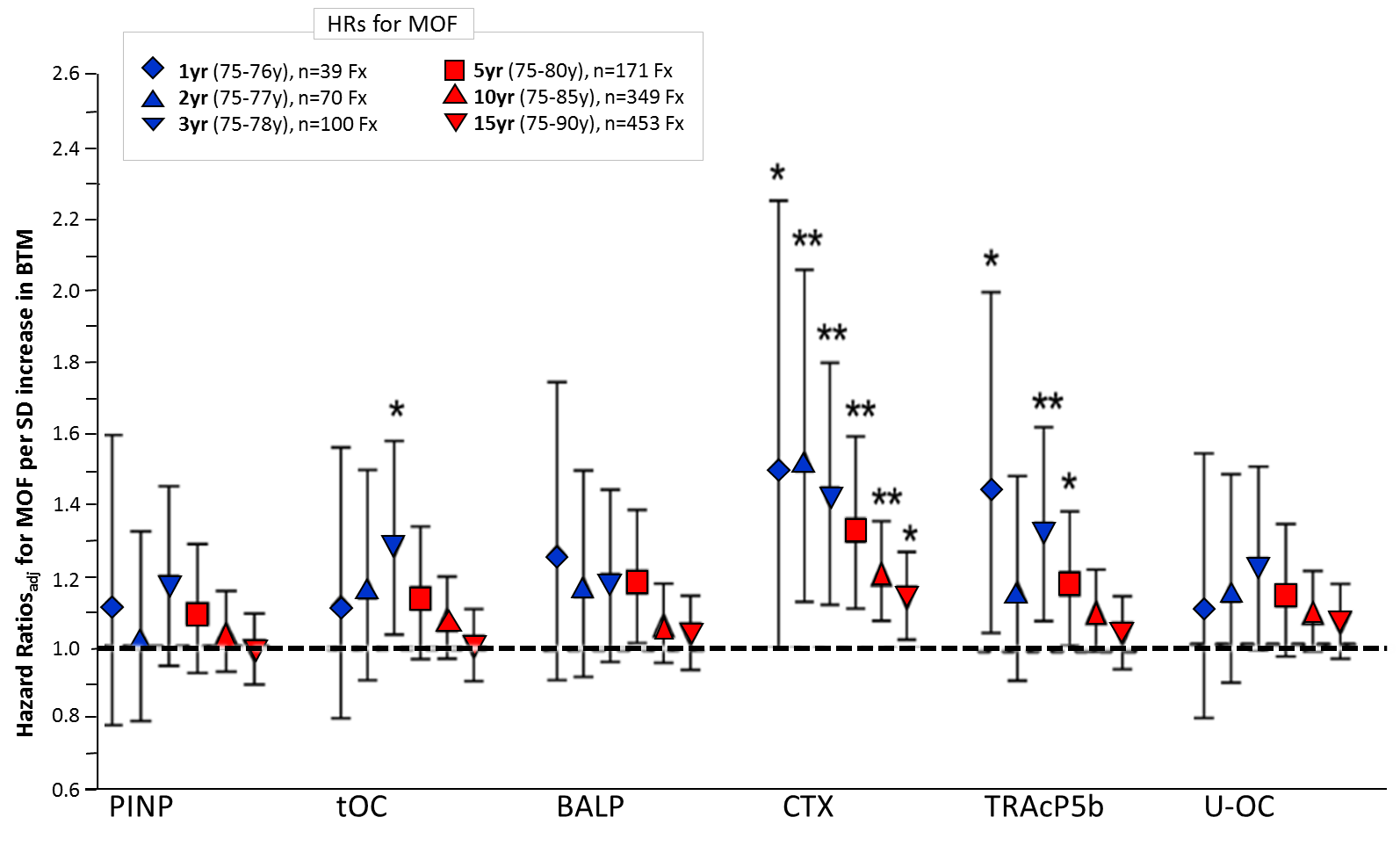
**

**Supplemental Figure 4.** **Hazard ratios for major osteoporotic fracture.** Hazard Ratios (HR) per SD increase in baseline BTM Z-score (95% CI) are presented for 1, 2, 3, 5, 10 and 15-year follow-up for each BTM, adjusted for baseline smoking, bisphosphonate use and prior osteoporotic fracture. Nominally significant HRs are marked with asterisks (*p<0.05, **p<0.01).
